# Identification of pyroptosis-related subtypes and establishment of prognostic model and immune characteristics in asthma

**DOI:** 10.3389/fimmu.2022.937832

**Published:** 2022-07-28

**Authors:** Fan Yang, Tieshan Wang, Peizheng Yan, Wanyang Li, Jingwei Kong, Yuhan Zong, Xiang Chao, Weijie Li, Xiaoshan Zhao, Ji Wang

**Affiliations:** ^1^ College of Traditional Chinese Medicine, Beijing University of Chinese Medicine, Beijing, China; ^2^ National Institute of Traditional Chinese Medicine (TCM) Constitution and Preventive Medicine, Beijing University of Chinese Medicine, Beijing, China; ^3^ Beijing Research Institute of Chinese Medicine, Beijing University of Chinese Medicine, Beijing, China; ^4^ College of Pharmacy, Shandong University of Traditional Chinese Medicine, Jinan, China; ^5^ Department of Clinical Nutrition, Chinese Academy of Medical Sciences - Peking Union Medical College, Peking Union Medical College Hospital, Beijing, China; ^6^ College of Traditional Chinese Medicine, Shandong University of Chinese Medicine, Jinan, China; ^7^ School of Chinese Medicine, Southern Medical University, Guangzhou, China

**Keywords:** pyroptosis-related genes, asthma, prognostic model, immune cell, DPP4

## Abstract

**Background:**

Although studies have shown that cell pyroptosis is involved in the progression of asthma, a systematic analysis of the clinical significance of pyroptosis-related genes (PRGs) cooperating with immune cells in asthma patients is still lacking.

**Methods:**

Transcriptome sequencing datasets from patients with different disease courses were used to screen pyroptosis-related differentially expressed genes and perform biological function analysis. Clustering based on K-means unsupervised clustering method is performed to identify pyroptosis-related subtypes in asthma and explore biological functional characteristics of poorly controlled subtypes. Diagnostic markers between subtypes were screened and validated using an asthma mouse model. The infiltration of immune cells in airway epithelium was evaluated based on CIBERSORT, and the correlation between diagnostic markers and immune cells was analyzed. Finally, a risk prediction model was established and experimentally verified using differentially expressed genes between pyroptosis subtypes in combination with asthma control. The cMAP database and molecular docking were utilized to predict potential therapeutic drugs.

**Results:**

Nineteen differentially expressed PRGs and two subtypes were identified between patients with mild-to-moderate and severe asthma conditions. Significant differences were observed in asthma control and FEV1 reversibility between the two subtypes. Poor control subtypes were closely related to glucocorticoid resistance and airway remodeling. BNIP3 was identified as a diagnostic marker and associated with immune cell infiltration such as, M2 macrophages. The risk prediction model containing four genes has accurate classification efficiency and prediction value. Small molecules obtained from the cMAP database that may have therapeutic effects on asthma are mainly DPP4 inhibitors.

**Conclusion:**

Pyroptosis and its mediated immune phenotype are crucial in the occurrence, development, and prognosis of asthma. The predictive models and drugs developed on the basis of PRGs may provide new solutions for the management of asthma.

## Introduction

Asthma is a respiratory disease characterized by chronic inflammation of airways. Its prominent features include airway hyperresponsiveness and recurrent attacks. Although asthma has been extensively investigated, pathogenesis requires further investigation and existing studies mainly focus on the airway inflammatory mechanism, immune regulation mechanism, neuromodulatory mechanism, and genetic and environmental factors (1). Severe asthma refers to the type that requires step 4 or 5 treatment, as recommended by Global Initiative for Asthma (GINA). This kind of asthma is more difficult to control than its mild-to-moderate counterpart (2), thereby reducing the quality of life and increasing the mortality of patients in this group. A variety of immune cells are involved throughout the course of asthma. The GINA guidelines indicated that monoclonal antibody-based immunotherapy is recommended for patients with severe asthma (3). Although severe asthma is mostly characterized by type-I immunity, drugs typically used are generally under target type-I immunity and sometimes unavailable (4). Therefore, cutting off the progression of mild-to-moderate to severe asthma cases is still the key to asthma management given the disease characteristics and health economic expenditure because sensitive assessment tools are required in identifying early mild-to-moderate asthma patients and the development of drugs to treat asthma from other pathophysiological perspectives and help patients achieve clinical control (5).

Cell pyroptosis has gradually become an important research direction in the exploration of asthma pathogenesis. Pyroptosis is a new mode of programmed cell death that has been confirmed in recent years. Pyroptosis is characterized by the change of osmotic pressure inside and outside the cell due to the perforation of activated gasdermin D (GSDMD) in the cell membrane and flow of extracellular material into the cell that result in its final rupture and release of massive proinflammatory factors (6). Gasdermin B (GSDMB) intervenes in various physiological and pathological processes, such as inflammation, coagulation, and cell differentiation, by regulating cell pyroptosis. Moreover, gene polymorphism of GSDMB is highly correlated with the pathogenesis of asthma (7). The expression of GSDMB in airway epithelial cells of patients with asthma is upregulated. Cell pyroptosis induced by the GSDMB protein can be eliminated when deleting the entire exon 6 from the splicing variant rs11078928 of the GSDMB gene to reduce the risk of asthma (8). Additionally, immune cells mediate the progression of asthma through cell pyroptosis. Activated caspase-11 induces pyroptosis, alveolar macrophages from patients with asthma exhibit increased expression of caspase-4 (human homologue of caspase-11), and prostaglandin E2 can exert protective effects against allergic airway inflammation by inhibiting caspase-11-/caspase-4-dependent pyroptosis in mouse and human macrophages (9). Although existing studies have initially reported the role of pyroptosis and pyroptosis-related genes (PRGs) in the development and progression of asthma, research on expression differences of PRGs between mild-to-moderate and severe asthma cases is still lacking and the role of cell pyroptosis in immune responses in airway epithelial tissues remains unclear. Moreover, studies that combine the situation of asthma control and immune imbalance as well as comprehensively analyze the role of PRGs in asthma are limited.

We excluded key PRGs related to the progression and control of asthma using machine learning method, compared the differences of immune cell infiltration under different PRG expression patterns, and established the relationship between key PRGs and immune cells in this study. A pyroptosis-related prognostic model was constructed to predict the control of asthmatic patients, and animal experiments were performed to verify the effectiveness of the model and screen potential therapeutic agents for high-risk patients.

## Materials and methods

### Data source

The microarray datasets GSE89809 and GSE104468 are downloaded from the GEO database (https://www.ncbi.nlm.nih.gov/geo/) (10, 11). The “*limma*” package was used to screen differentially expressed genes (DEGs) (12), and *P* < 0.05 and *|*log2 FC*|* > 0.585 were considered statistically significant. The 227 pyroptosis-related genes are from the Genecards (https://www.genecards.org/) website. The site uses GeneCards Inferred Functionality Scores (GIFtS) to annotate each relevant gene and generate a correlation score. In this study, pyroptosis was used as the keyword to search related genes, and 227genes with GIFtS > 30 were included for follow-up study.

### Classification and functional enrichment analysis of PRG-related subtypes in asthma

Unsupervised cluster analysis was carried out according to the difference of PRG expression between mild-to-moderate and severe asthma cases to identify various subtypes of asthma (13, 14). A consensus clustering algorithm was used to evaluate the cluster numbers and robustness. The R package “*ConsensuClusterPlus*” was applied to implement these steps for 1000 iterations to guarantee the robustness of classification (15). Weighted gene coexpression network analysis for specific subtypes was performed with the R package “*WGCNA*.” (16, 17) We first determined the appropriate soft threshold power to achieve a scale-free topology. Hierarchical clustering was then performed to identify modules, and a phylogenetic cluster map was constructed to divide similar gene expression levels into different modules. The correlation between the phenotype composed of various clinical information and each module was evaluated using Pearson correlation analysis. The module with the maximum correlation with the trait was selected for subsequent analysis. The ChEA3 database (https://maayanlab.cloud/chea3/) includes a large number of independently published ChIP-seq data and integrates transcription factor coexpression data on the basis of RNA-seq data (18). The transcriptional regulatory network of key modules is predicted from this database. Gene set enrichment analysis (GSEA) was performed on the gene expression matrix through the “*clusterProfiler*” package, and “*c2.cp.kegg.v7.0.symbols.gmt*” was selected as the reference gene set. We used the “*clusterProfiler*” and “*ggplot2*” packages to perform Gene Ontology (GO) and Kyoto Encyclopedia of Genes and Genomes (KEGG) enrichment analyses on DEGs, respectively (19, 20).

### Screening, expression regulation, and immune cell infiltration analyses of diagnostic markers associated with pyroptosis subtypes

We used least absolute shrinkage and selection operator (LASSO) logistic regression to perform feature selection of screening diagnostic markers for asthma (21). LASSO is a popular algorithm, which was extensively utilized in medical studies (22–26). The LASSO algorithm was applied with the “*glmnet*” package (27). The regulatory prediction of noncoding RNAs for diagnostic markers was first analyzed using databases, including RNA22, miRDB, and RNAInter, to select miRNAs with intersections (28). The literature was then reviewed to select miRNAs with biological roles in asthma for further analysis. miRNet 2.0 database (www.mirnet.ca/miRNet/home.xhtml) and starBase (http://starbase.sysu.edu.cn/)) are utilized to predict the target lncRNA of miRNA and finally establish the ceRNA network (29, 30). The degree of immune cell infiltration in the airway epithelium was assessed using the CIBERSORT algorithm to determine the abundance of 22 immune cell subsets, and differences in immune cell infiltration between groups were visualized through the “*ggplot2*” package (31, 32). Finally, Spearman correlation analysis was performed on all immune cells and diagnostic markers, and the positive or negative correlation between them was determined with the “*ggstatsplot*” package.

### Construction of a risk signature associated with asthma control

The univariate Cox regression model was utilized to select genes related to the control of patients with asthma. *P* < 0.05 was considered statistically significant. The optimal genome for constructing risk characteristics was screened using LASSO regression to establish the risk score as follows: Risk score = (expr_gene1_ × Coef_gene1_) + (expr_gene2_ × Coef_gene2_) + … + (expr_genen_ × Coef_genen_) (33). Patients with asthma were divided into low- and high-risk groups according to the median risk score. Principal component analysis (PCA) and t-distributed stochastic neighbor embedding (t-SNE) were used to evaluate the classification accuracy of risk scores. In addition, a Cox proportional hazards regression model was applied to identify the risk score as an independent predictor, and Kruskal test was selected to compare infiltrating immune cell abundance scores in two different risk patterns.

### Identification of small-molecule therapeutic drugs that block the progression of asthma

The Broad Institute Connectivity Map (cMAP) database (https://portals.broadinstitute.org/cmap) was used to identify small-molecule drugs associated with asthma progression. Gene sets with log2FC > 0.585 were entered into the cMAP database for enrichment analysis to identify drug candidates (34). The PubChem database (https://pubchem.ncbi.nml.gov) was utilized to extract details of small molecules and obtain their 3D structures. Molecular docking was then carried out, and the predicted crystal structure information of the target protein (human source) was searched in the PDB (https://www.rcsb.org/) database. The receptor protein was pretreated with dehydration and hydrogenation using AutoDockTool software, and the molecular docking between the target protein and small molecular drugs was carried out Vina software to predict the binding ability of the two proteins. A negative binding energy value and an absolute value greater than 5 kcal/mol demonstrate significant binding possibilities.

### Animal experiment

BALB/c mice (female, 17–20 g, 6–8 weeks old) were obtained from Beijing Vital River Laboratory Animal Technology Co., Ltd. in China. The mice were kept under specific pathogen-free conditions in Beijing University of Chinese Medicine. All mice were kept in a controlled room (25°C ± 1°C, 45%–60% humidity). All animal studies were conducted in accordance with the institutional animal care regulations of Beijing University of Chinese Medicine and AAALAC and IACUC guidelines. The allergic asthma model of mice was established through OVA sensitization and atomization inhalation stimulation. Briefly, mice were intraperitoneally injected with 2 mg of OVA (Sigma–Aldrich, Cat#A5503) mixed with 2 mg of Imject™ Alum Adjuvant (Invitrogen, Cat#77161) and PBS on days 0 and 14. The challenge phase was from the 21st day to the 25th day after injection, and the mice were atomized with 1% OVA for 30 minutes. The animals were then sacrificed, and lung tissues were snap-frozen in liquid nitrogen and stored in a freezer at −80°C for further analysis.

### Western blot

Rabbit anti-BNIP3 antibody (bs-4239R) was purchased from Biosynthesis Biotechnology Inc. (Beijing, China). Horseradish peroxidase-labeled goat antirabbit IgG antibodies (GB23303) was obtained from Wuhan Service Bio Technology Co., Ltd. We extracted the protein from lung tissues according to the manufacturer’s instructions after obtaining the total protein from lung tissues using radio immune precipitation assay (RIPA) lysis buffer containing 0.1% phenyl methyl sulfonyl fluoride (PMSF). We used the BCA protein detection kit to measure the protein concentration. Equal amounts of protein samples were separated with sodium dodecyl sulfate–polyacrylamide gel electrophoresis (SDS-PAGE) and transferred to polyvinylidene fluoride (PVDF) membranes. Nonfat milk (5%) in TBST was incubated overnight with primary antibodies against BNIP3 (diluted 1:1,000) at 4°C after blocking. We incubated the sample using goat antirabbit secondary antibody for 1.5 h at room temperature and then washed it four times with TBST to form protein bands on the membrane with an enhanced chemiluminescence reagent. AlphaEase FC software was applied to detect the gray value of protein bands.

### Quantitative real-time polymerase chain reaction

mRNA was extracted from lung tissues using a universal RT-PCR kit (Solarbio Science & Technology Co., Ltd., Shanghai, China) following the manufacturer’s instructions. Samples were treated with DNase and then purified using an RNeasy kit (Qiagen, Hilden, Germany). Glyceraldehyde-3-phosphate dehydrogenase (GAPDH) was utilized as the internal reference. PCR primer sequences include the following: SLC4A1: forward primer: 5’-CTTCTTCTCGTTCTGTGAAAGCAAT-3’, reverse primer: 5’-CATAAGTCTGTTGTAGTGGGTAGTCC-3’, SERPINB2: forward primer: 5’-AGGTGAAATCCCAAACCTGCTAC-3’, reverse primer: 5’-CACGGAAAGGATAAAGCCCAT-3’, BNIP3: forward primer: 5’-TTCCAGCCTCCGTCTCTATTTA-3’, reverse primer: 5’-AATCTTCCTCAGACAGAGTGCTG-3’, TAAR9: forward primer: 5’-TTACACGGGAGCCAATGAGG-3’, reverse primer: 5’-TGGCTGTACCCTCTATCTTCCTA-3’, GAPDH: forward primer: 5’-CCTCGTCCCGTAGACAAAATG-3’, and reverse primer: 5’-TGAGGTCAATGAAGGGGTCGT-3’.

### Statistical analysis

Data were shown as mean ± standard deviation (SD). The differences between the two groups were evaluated by independent sample t-test and nonparametric test. A *P* < 0.05 was considered statistically significant. Statistical analyses and figures were obtained using IBM SPSS Statistics 23.0 (IBM SPSS Software, NY, USA) and GraphPad Prism Version 8.0 (GraphPad Software, San Diego, CA, USA).

## Results

### Landscape of PRGs between mild-to-moderate and severe asthma samples

**Figure 1:**
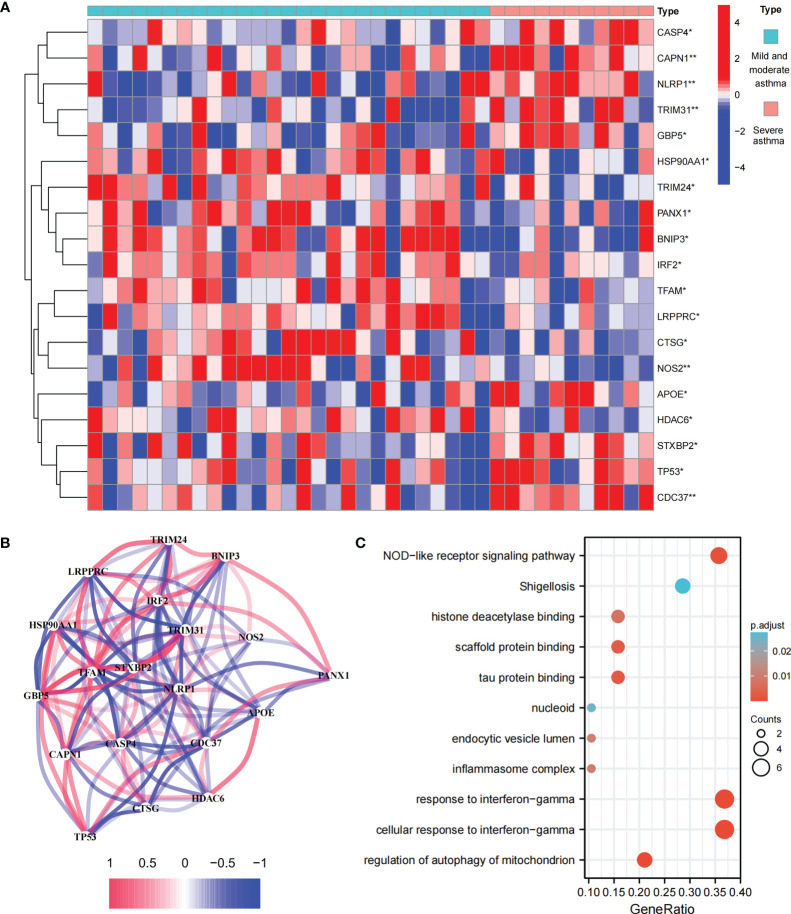
Expression and biological function of PRG in mild to moderate asthma and severe asthma. **(A)** 19 differentially expressed PRGs between mild to moderate and severe asthma. **(B)** Co-expression network of 19 PRGs. **(C)** GO and KEGG enrichment analysis of differentially expressed PRG. *P < 0.05 , **P < 0.01.

This study involved 227 PRGs. Nineteen differentially expressed PRGs were included in the mild-to-moderate and severe asthma samples, among which CAPN1, NLRP1, TRIM31, NOS2, and CDC37 showed the highest difference (*P*<0.01, **Figure 1A**). The analysis of the coexpression relationship of 19 PRGs demonstrated that a close correlation exists between gene expression levels (**Figure 1B**). GO and KEGG analyses demonstrated that differentially expressed PRGs are mainly involved in biological processes and signaling pathways, such as cellular response to interferon-gamma and NOD-like receptor signaling pathway (**Figure 1C**). These findings suggested that differences likely exist in the cell pyroptosis level in the airway epithelium of mild-to-moderate and severe asthma patients and PRGs may be involved in the evolution of clinical symptoms or disease course of patients through mediated inflammatory processes.

### Differentially expressed PRGs between mild-to-moderate and severe asthma cases are divided into two subtypes

**Figure 2:**
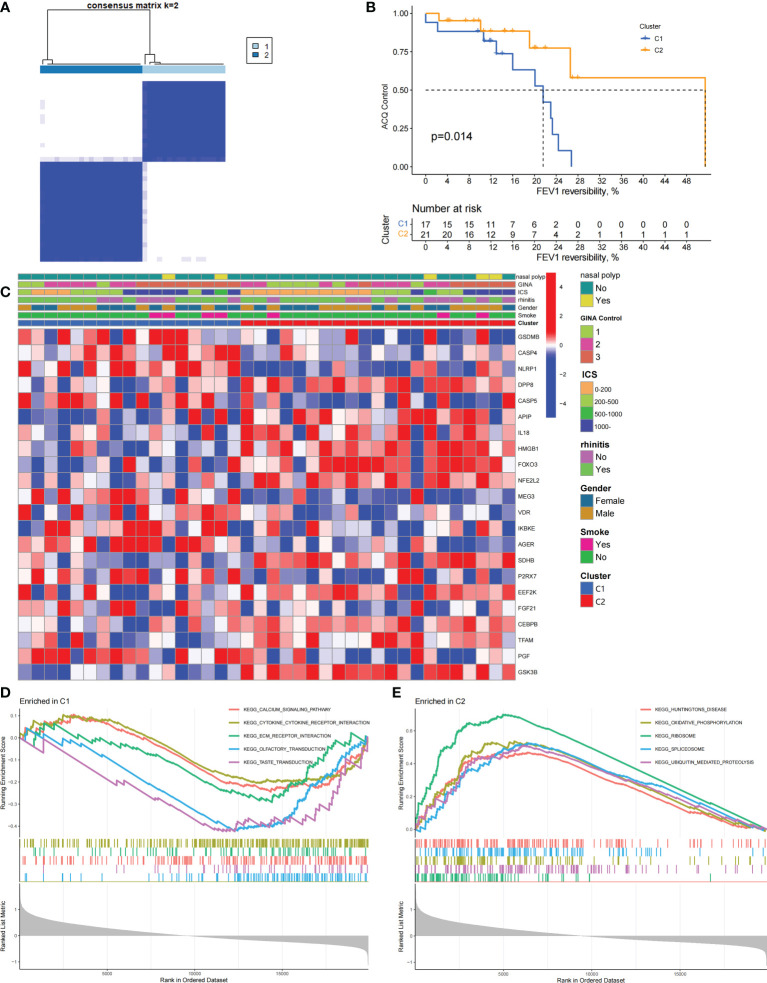
Two different pyroptosis-related subtypes identified in asthma by unsupervised clustering of 19 PRGs. **(A)** Heatmap of the matrix of co-occurrence proportions for asthma samples. **(B)** Kaplan–Meier curves of different gene subtypes ACQ Control and FEV1 reversibility. **(C)** Composite heatmap showing the relationship between the expression levels of 22 PRGs and clinical features. **(D, E)** GSEA analysis of C1 and C2 subtypes.

We performed an unsupervised consensus cluster analysis of asthma samples on the basis of the expression of 19 PRGs to investigate the role of PRGs in asthma. According to the cumulative distribution function curve, k=2 is selected as the best number of clusters (**Figure 2A**). This finding indicated that two pyroptosis-related molecular subtypes (C1 and C2) are observed in asthma. FEV1 reversibility, a marker of airway hyperresponsiveness (AHR), is positively correlated with the severity of asthma (35, 36). The asthma control questionnaire (ACQ) is a widely used standardized questionnaire of asthma control that can stably and effectively evaluate the degree of control in patients (37–39). Joint analysis of the two major key clinical features of ACQ control and FEV1 reversibility revealed that patients with the C1 subtype present severely poor asthma control (*P* = 0.014, **Figure 2B**). Twenty-two differentially expressed PRGs were observed between C1 and C2 subtypes. The analysis of relevant clinical characteristics showed that the C1 subtype (0–200 μg: three cases, 1000 μg or more: seven cases) is higher than the C2 subtype (0–200 μg: 10 cases, 1000 μg or more: five cases) when ICS is used. This finding suggested that poor asthma control in patients with the C1 subtype may be due to a certain degree of glucocorticoid resistance (**Figure 2C**). GO and KEGG enrichment analyses of DEGs among subtypes were performed, and biological processes, such as ribosomal translation and threonine-type endopeptidase activity, were significantly enriched (**Figure S1**). GSEA enrichment analysis showed that the difference of main signal pathways between C1 and C2 subtypes (**Figures 2D, E**) is that the C1 subtype is mainly enriched in the calcium signaling pathway, cytokine–cytokine receptor interaction, and other pathways closely related to allergy and asthma. These results suggested that PRGs involved in the exacerbation of asthma present a satisfactory classification function that can be used can be used in clinical practice for the detection and intervention of PRGs.

### Biological characteristics of the C1 subtype

**Figure 3:**
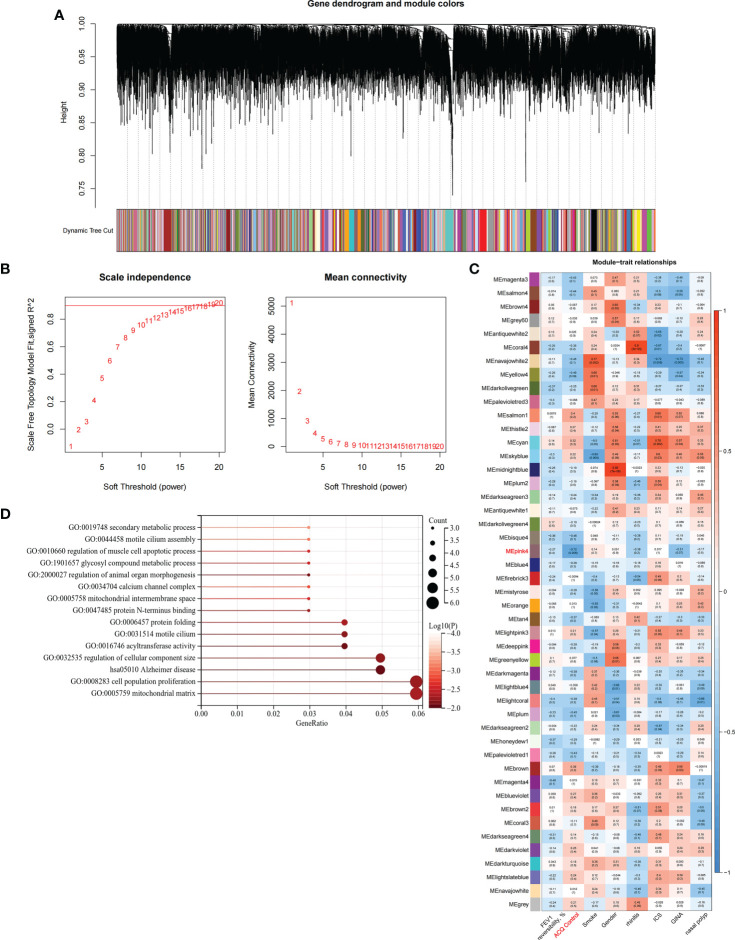
Identification and functional enrichment analysis of asthma control related genes in C1 subtype. **(A)** WGCNA of the C1 subtype to obtain a cluster dendrogram of coexpressed genes. **(B)** Analysis of the scale free ft index and analysis of the mean connectivity for various soft – theresholding powers. **(C)** Module – trait relationships for C1 subtypes. Each module contains the corresponding correlation and *P*-value. **(D)** GO and KEGG functional enrichment analysis of genes in MEpink4 module.

We constructed scale-free networks and determined optimal soft thresholds on the basis of clinical features to explore related genes that affect asthma control in C1 subtype patients (**Figures 3A, B**). The TOM matrix was utilized to detect the gene module and analyze the correlation with clinical characteristics. The results showed that the MEpink4 module presents the maximum correlation with asthma control (cor = −0.72, *P* = 0.006). Hence, this module was selected for subsequent analysis (**Figure 3C**). Genes within the same cluster often share common transcription factors. We predicted and analyzed the transcription factors of genes in the MEpink4 module and visualized the mutual regulatory relationship between the top 10 transcription factors in the mean rank (**Figure S2**). The association of these transcription factors with asthma was partially demonstrated. For example, SNPs of ZBTB10 affect the asthma risk through the cis-regulation of its genes (40). Functional enrichment analysis of genes in the MEpink4 module showed their primary involvement in biological processes and cellular components related to allergic reactions and airway remodeling, such as motile cilium assembly, regulation of muscle cell apoptotic process, and calcium channel complex, which are closely related to glucocorticoid resistance and poor control of the C1 subtype (**Figure 3D**).

### Determination of pyroptosis-related diagnostic markers among subtypes and their correlation with immune cells

**Figure 4:**
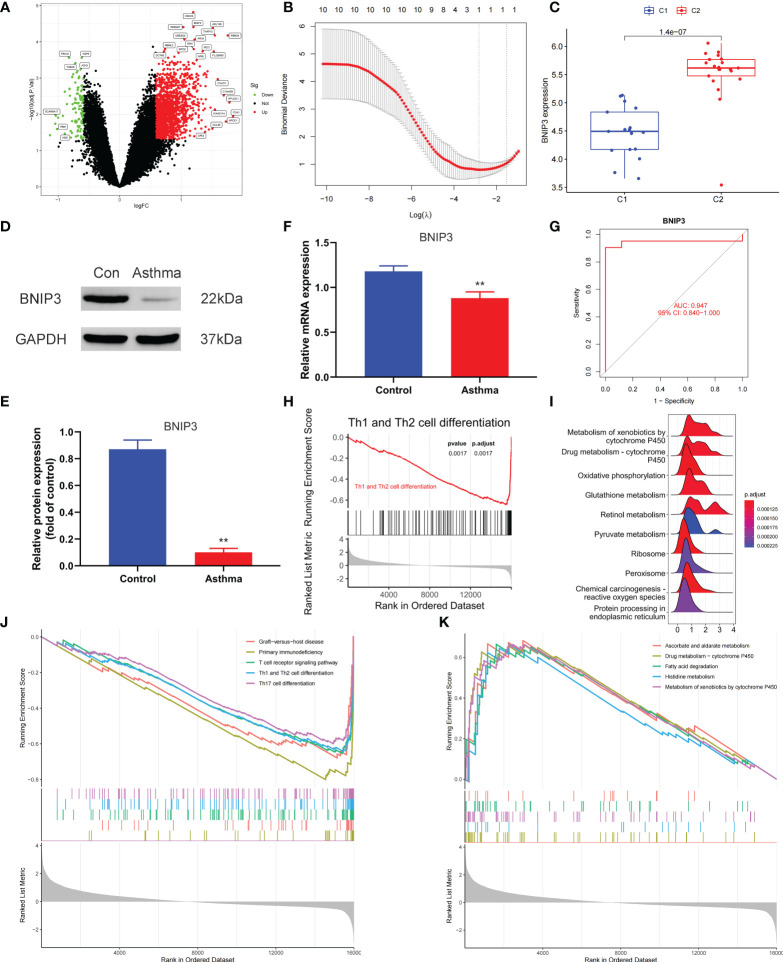
Screening of pyroptosis-related diagnostic markers. **(A)** Volcano map of DEGs. red represents up-regulated differential genes, black represents no significant difference genes, and green represents down-regulated differential genes. **(B)** LASSO logistic regression algorithm to screen diagnostic markers. **(C)** Difference of BNIP3 gene expression between C1 and C2 subtypes. **(D, E)** Differential expression of BNIP3 between normal and asthmatic mice. **(F)** Differential expression of BNIP3 mRNA between normal and asthmatic mice. **(G)** ROC curve for verifying the efficacy of BNIP3 gene diagnosis. **(H)** Enrichment difference of Th1 and Th2 cell differentiation pathway between BNIP3 high expression group and low expression group. **(I)** Functional enrichment analysis of DEGs between BNIP3 high expression group and low expression group. **(J, K)** GSEA enrichment analysis of DEGs between BNIP3 high expression group and low expression group. **P < 0.01.

**Figure 5:**
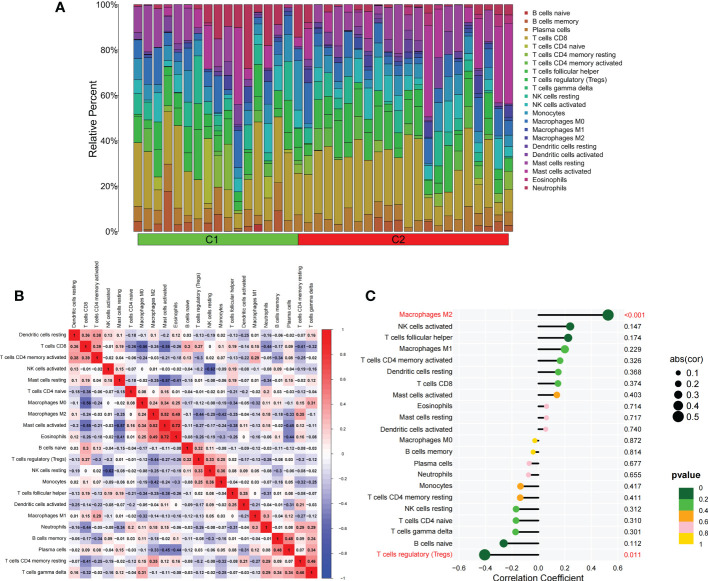
The difference of immune cell infiltration among subtypes. **(A)** The histogram of the infiltration ratio of 22 kinds of immune cells in C1 group and C2 subtype samples. **(B)** The correlation among 22 kinds of immune cells. **(C)** The lollipop diagram shows the correlation between BNIP3 gene and 22 kinds of immune cells, and red marks indicate *P* < 0.05.

**Figure 6:**
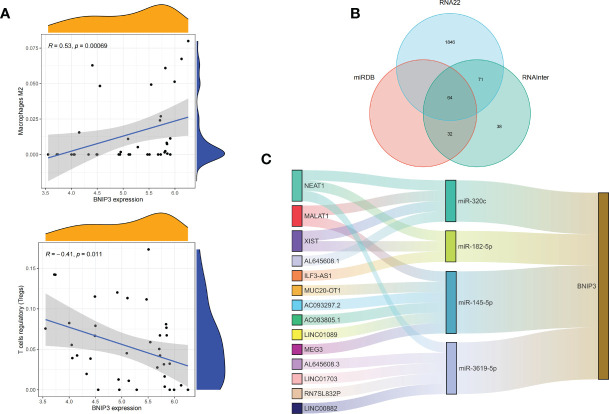
Correlation of BNIP3 with immune cell infiltration and its gene expression regulatory network. **(A)** Mountain diagram showing the correlation between the BNIP3 gene and immune cells. **(B)** The intersection of the prediction results of the three databases. **(C)** CeRNA networks involved in the regulation of BNIP3 gene expression.

We applied the LASSO logistic regression algorithm on the basis of DEGs and differentially expressed PRGs between C1 and C2 subtypes (**Figure 4A**) to identify the BNIP3 gene as a pyroptosis-related diagnostic marker for asthma (**Figure 4B**). Expression levels of BNIP3 in C1 and C2 subtypes were significantly different *(P*<0.01), and expression levels of the C1 subtype were lower than those of the C2 subtype (**Figure 4C**). This finding suggested that the low expression of BNIP3 may lead to poor asthma control. Animal experiments confirmed that the content of BNIP3 protein in the lung tissue of the asthma mouse model is significantly downregulated (**Figures 4D, E**) and the BNIP3 mRNA shows the same trend (*P*<0.01, **Figure 4F**) compared with those of normal mice. Meanwhile, the ROC curve showed the satisfactory classification performance of BNIP3 for C1 and C2 subtypes (AUC: 0.947, **Figure 4G**). Samples were divided into two groups with high- and low-BNIP3 expression levels for single-gene GSEA analysis to explore the role of BNIP3 in asthma. The results showed that a significant difference exists in the enrichment degree of cell differentiation of Th1 and Th2 in the key pathway of asthma between the two groups (*P* = 0.0017, **Figure 4H**) biological processes, such as metabolism of xenobiotics by cytochrome P450 and glutathione metabolism, are primarily involved (**Figure 4I**). Notably, the low expression of BNIP3 was highly correlated with the T cell receptor signaling pathway, cell differentiation of Th1 and Th2, Th17 cell differentiation, and other immune processes (**Figsures 4J, K**). We quantified the level of immune cell infiltration with the composition of 22 immune cells in all samples between the two subtypes shown in histograms to assess the immune landscape of C1 and C2 subtypes (**Figure 5A**). Statistical differences in immune cell infiltration between the two subtypes showed that infiltration of M2 macrophages is less in the C1 subtype compared with that in the C2 subtype (*P*=0.01, **Figure S3**). The relationship between immune cells is illustrated in the thermogram in **Figure 5B**. Several immune cells closely related to asthma showed a strong correlation. M2 macrophages were positively correlated with activated mast cells and eosinophils, and plasma cells were negatively correlated with activated mast cells and eosinophils. Spearman correlation analysis tested the correlation between BNIP3 and immune cells (**Figure 5C**) and revealed that it was significantly positively correlated with M2 macrophages (*R*=0.53, *P*=0.00069) and significantly negatively correlated with regulatory T cells (*R*=−0.41, *P*=0.011, **Figure 6A**). The noncoding RNA regulatory mechanism of BNIP3 was subsequently explored, and 64 miRNAs were predicted from three databases (**Figure 6B**), of which four miRNAs presented functions in asthma. Finally, a ceRNA network, including 14 LncRNAs and four miRNAs, was constructed (**Figure 6C**). However, their regulatory relationship and biological significance in asthma still need further investigation.

### Establishment of a prognostic model, immunological characteristics, and drug prediction based on asthma control

**Figure 7:**
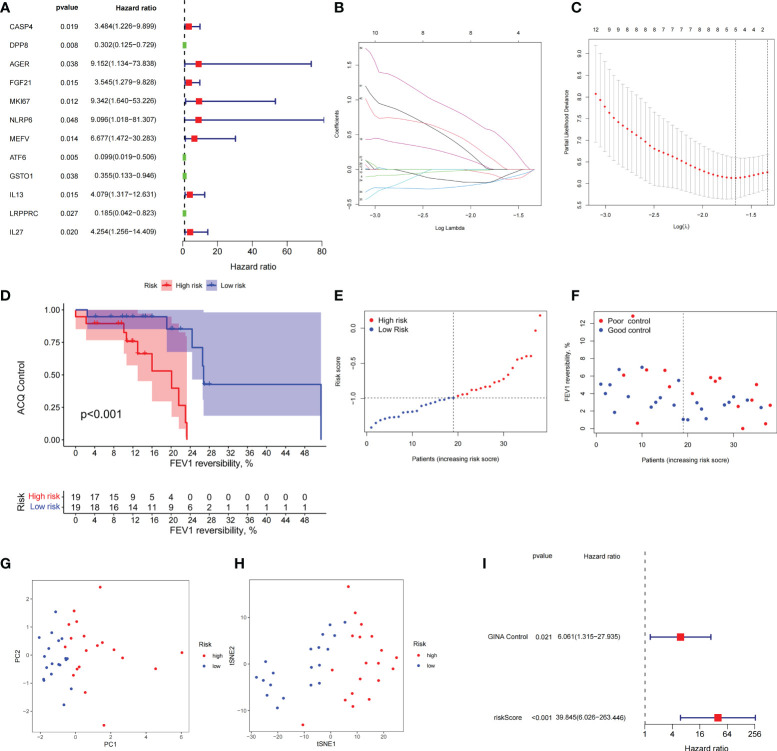
The prognostic model constructed by differentially expressed genes from different pyroptosis patterns can distinguish between high- and low-risk patients with asthma. **(A)** DEGs associated with asthma control status. Red and green colors represent high- and low-risk genes, respectively. **(B, C)** Distribution of LASSO coefficients for DEGs. Tenfold cross-validation for tuning parameter selection in the LASSO regression. Dotted vertical lines are drawn at the optimal values by minimum criteria and 1 − SE criteria. **(D)** Kaplan–Meier curves of ACQ control and FEV1 reversibility in patients with different risk groups. **(E)** P-score distribution of patients with different risks. **(F)** The distribution of FEV1 reversibility and asthma control among different risk samples. **(G, H)** PCA and t-SNE was used to determine whether the samples could be grouped correctly based on the P-score. **(I)** Multivariate Cox regression analysis of P-score was shown by forest plot.

**Figure 8:**
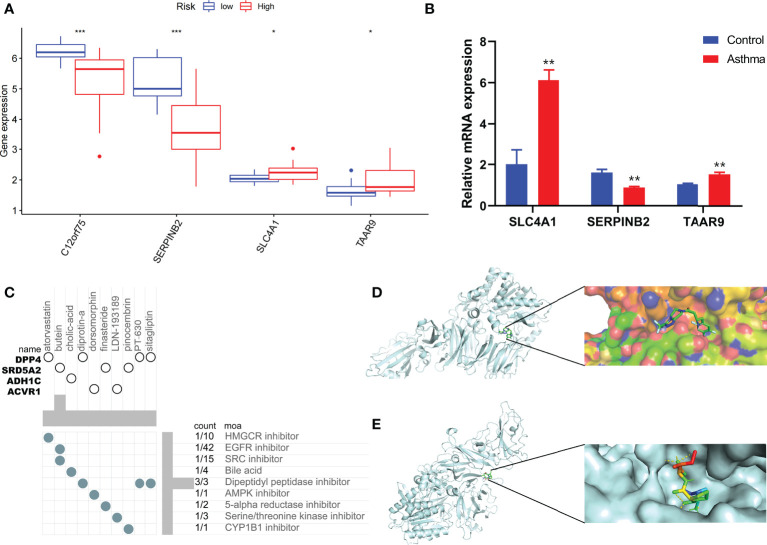
Immune status of patients at different risks and prediction of potential therapeutic drugs. **(A)** The expression differences of P-score constituent genes among different risk groups. **(B)** The difference of expression of P-score constituent genes between normal mice and asthmatic mice. **(C)** Correlation between potential therapeutic drugs and corresponding targets. **(D)** Binding conformation of DPP4 and diprotin-A (binding energy = −6.4 kcal/mol). **(E)** Binding conformation of DPP4 and sitagliptin (binding energy = −7.8 kcal/mol). *P < 0.05, **P < 0.01, ***P < 0.001.

Combined with clinical data, we identified 12 PRGs that can predict asthma control (**Figure 7A**). LASSO Cox regression analysis was performed on DEGs between subtypes of different cell pyroptosis levels to obtain a prediction model consisting of four genes (**Figures 7B, C**). The composition of the model is as follows: risk score = (−0.131 × C12orf75 expression level) + (−0.138 × SERPINB2 expression level) + (0.434 × SLC4A1 expression level) + (0.029 × TAAR9 expression levels). This model named P-score can be used to divide patients into high- and low-risk groups on the basis of the median cut-point value of the score. The results showed that the rate of asthma control level in the high-risk group declines faster than that in the low-risk group with the increase of FEV1 reversibility (*P* < 0.001, **Figure 7D**). Joint analysis of the patients’ p-score, FEV1 reversibility, and asthma control revealed that patients with poor control demonstrate high p-score and FEV1 reversibility (**Figures 7E, F**). PCA and t-SNE analyses were performed on gene expression data to test the classification ability of the P-score. The results showed a clear distribution difference between the two groups (**Figures 7G, H**). Furthermore, the multivariate regression analysis indicated that the P-score is an independent risk factor for asthma control (HR = 39.845, *P* < 0.001) (**Figure 7I**). We then explored the expression levels of four genes in the P-score of high- and low-risk groups and demonstrated that C12orf75 and SERPINB2 are downregulated while SLC4A1 and TAAR9 are upregulated in the high-risk group (**Figure 8A**). PCR analysis of SERPINB2, SLC4A1, and TAAR9 genes, which also exist in the mouse model of asthma, was conducted to verify the accuracy of the results. The findings showed that the level of SERPINB2 mRNA in the model of asthma is downregulated while that of SLC4A1 mRNA and TAAR9mRNA is upregulated compared with those of normal mice (**Figure 8B**). In order to verify the value of P-score, we used GSE104468 as the verification set to test the diagnostic efficacy of the three core genes. It is found that the AUC values of SERPINB2, SLC4A1, TAAR9 in patients with asthma and normal people are 0.854, 0.819 and 0.688, respectively (**Figure S4**). These conclusions suggested that the P-score can effectively predict the control status of asthma patients. We explored its immune cell abundance and biological processes using ssGSEA and GO analyses to understand the differences in immune microenvironment and biological functional characteristics among different degrees of risk groups mediated by cell pyroptosis. Relative to those in the low-risk group, the degree of B and Th1 cell infiltration was higher, and the degree of NK cell infiltration was lower in the high-risk group (*P*<0.05, **Figure S5**). Notably, DEGs between high- and low-risk groups were mainly concentrated in extracellular vesicle-related biological processes. This finding suggested that cell pyroptosis in asthma may be closely related to the occurrence of extracellular vesicles and intercellular communication (**Figure S6**). Finally, 10 small-molecule drugs targeting high-risk asthma were screened using the cMAP database, and their correlation with DPP4 targets was significant (**Figure 8C**). Sitagliptin and diprotin-A were selected for molecular docking with DPP4 to confirm the binding ability between the drug and the target. The results showed that affinities between them are all less than −5 kcal/mol. The molecular docking patterns of the two molecules are shown in **Figures 8D, E** confirmed that the drug and the target show significant binding ability, which may play a potential therapeutic role in asthma.

## Discussion

Asthma is a common chronic disease. Existing treatment goals for asthma are mainly to prevent exacerbations and reduce the risk of further exacerbations. However, its heterogeneity increases the difficulty in achieving these goals. Th2-driven asthma is a common clinical manifestation typically characterized by elevated levels of type-2 inflammatory factors, serum IgE, and blood eosinophils. However, these indicators are ineffective diagnostic markers for differentiating asthma control (41). By contrast, the relatively rare Th1-driven and severe asthma cases caused by various factors are usually poorly controlled, with limited choice of drugs and lacking predictive tools. Moreover, early identification of Th1-driven and severe asthma in patients at risk of poor control is challenging due to the unclear pathogenesis of asthma (42). Therefore, conducting an in-depth discussion on the basis of clinical and transcriptome characteristics of patients with different disease courses demonstrates high clinical and practical significance.

Although the pathogenesis and immune characteristics of asthma based on cell pyroptosis have been investigated, the role of pyroptosis in asthma remains unclear. Recent reports have shown that NLRP3-related inflammatory response is a key driver of asthma pathogenesis and the allergen Der f1 induces bronchial asthma by mediating caspase-1-dependent pyroptosis through the NLRP3 pathway (43, 44). Furthermore, the expression of canonical PRGs, such as GSDMB, caspase-4, and NLRP1, was upregulated in the poorly controlled asthma subtype. Therefore, the inhibition of pyroptosis in bronchial epithelial cells can reduce the inflammatory response of asthma. GSDMB can be cleaved into several short fragments when coexpressed with caspase-1, of which the N fragment (GSDMB-N) can induce significant cell pyroptosis (8). This phenomenon may explain why different GSDMB genotypes are associated with various phenotypes of adult asthma (45). Chromosome 17q12-q21 is the most replicated genetic locus in childhood asthma. The main mechanism is to regulate its SNPs to affect the abundance of GSDMB transcripts in airway epithelial cells and the functional properties of GSDMB proteins (46). The machine learning results of our study showed that BNIP3 is a diagnostic marker of different pyroptosis subtypes that affects the cell pyroptosis of cardiomyocytes and coronary artery endothelial cells by mediating caspase-3/GSDME and oxidative stress (47, 48). Note that the expression of BNIP3 was upregulated in properly controlled asthma subtypes but positively correlated with typical type-2 inflammatory cells, such as M2 macrophages, in subsequent immune infiltration analyses. This finding indicated that the asthma phenotype of poorly controlled groups may be different from type-2 inflammation in pyroptosis-based asthma typing. Moreover, among the various phenotypes of asthma, the expression of NLRP3, caspase-1, caspase-4, and other PRGs in neutrophil asthma significantly increases and NLRP3 inflammatory bodies can drive animal asthma models to produce glucocorticoid resistance (49, 50). Furthermore, asthma subtypes with upregulated NLRPs and caspase-1 exhibit high glucocorticoid use and poor asthma control. The consistency in these conclusions indicated that PRG-based typing methods show high potential in identifying immunophenotypes of patients with asthma and can be used to guide the use of glucocorticoid.

Although severe asthma will likely demonstrate poor control compared with mild-to-moderate asthma, poor control caused by gene expression differences, environmental stimulation, medication habits, infections, and other factors can occur in both mild-to-moderate and severe asthma cases (51). Therefore, we constructed the P-score model to predict the control profile of asthma by combining the differential expression levels of PRGs with clinical features. The role of the four genes that make up the P-score in asthma is also partially supported by previous studies. For example, meta-analysis based on the results of sputum transcriptome sequencing of asthmatic patients identified SERPINB2 as a potential signature gene associated with asthma pathogenesis and miR-34b as a key miRNA that regulates the SERPINB2 gene expression (52). Environmental pollutants, such as diesel engine exhaust particles, can promote the expression of the SERPINB2 gene in bronchial epithelial cells while inducing an asthma attack, and its expression level is significantly correlated with the severity of asthma (53, 54). Moreover, glucocorticoid treatment response is a crucial control for asthma accompanied by changes in the expression level of SERPINB2 (55). Although the P-score presents satisfactory risk prediction and discriminating ability, the association of the three other genes with asthma, except for SERPINB2, remains unverified. Hence, the mechanism of the P-score-predicting asthma control requires further experimentation. On the basis of DEGs of high-risk patients, the cMAP database identified targets and drugs with potential therapeutic value for asthma, among which DPP4 demonstrated the highest enrichment score as a drug action target. The level of DPP4 increases in airway epithelial cells of asthmatic patients, and its ability to promote airway smooth muscle cell proliferation *in vitro* implied that DPP4 is likely associated with airway remodeling in asthma (56). Interleukin 13 (IL-13) secreted by Th2 cells is associated with airway inflammation in asthma, and data based on transcriptome sequencing of asthmatic patients confirmed that a positive correlation exists between the DPP4 gene and IL-13 mRNA levels. This finding indicated that a DPP4 can play a potentially important role in asthma (57). Notably, the resulting DPP4 inhibitor sitagliptin predicted in this study can ameliorate airway inflammation in murine asthma models by downregulating inflammatory factors, such as IL-13. Therefore, these drugs may exert important effects on the treatment and control of asthmatic patients although they still need further investigations.

Asthma was successfully classified on the basis of the differential expression of PRGs between mild-to-moderate and severe asthma cases, and the significance of pyroptosis-mediated immunophenotype in the occurrence, development, and prognosis of asthma was systematically revealed in this study. The prognostic model can be used as a powerful tool in predicting asthma control. However, this study presents the following limitations. Existing asthma transcriptome sequencing datasets generally lack corresponding clinical data; hence, the number of patients that can be included in the study is small and the validation set is lacking. Moreover, further experiments are needed to provide new insights into the biological functions of other PRGs in asthma.

## Conclusions

We obtained pyroptosis-related genes associated with the progression of asthma in this study. We differentiated two pyroptosis-related subtypes and explored the relationship between key diagnostic markers and immune cells by assessing the expression levels of these genes. We also constructed a pyroptosis-related risk score model with acceptable predictive value and predicted the potential therapeutic drugs using this model. Our findings initially revealed the role of pyroptosis in asthma and promoted the improvement of treatment strategies.

## Data availability statement

The datasets presented in this study can be found in online repositories. The names of the repository/repositories and accession number(s) can be found in the article/**supplementary material**.

## Ethics statement

The animal study was reviewed and approved by experimental animal ethics subcommittee of Academic Committee of Beijing University of Traditional Chinese Medicine.

## Author contributions

FY designed and conducted the whole research. TW, PY, and WYL carried out animal experiments and molecular biological analysis. JK and YZ applied for the GEO dataset analysis of asthma. FY, XC, and WJL completed the data analysis and drafted the manuscript. XZ and JW revised and finalized the manuscript. All authors contributed to the article and approved the submitted version.

## Funding

This work was supported by the National Key R&D Program of China (2020YFC2003100, 2020YFC2003101), National Natural Science Foundation of China (No. 82174243, No. 81973715, No. 82004233), General project of Beijing Natural Science Foundation (No. 7202110), Innovation Team and Talents Cultivation Program of National Administration of Traditional Chinese Medicine (No. ZYYCXTD-C-202001).

## Conflict of interest

The authors declare that the research was conducted in the absence of any commercial or financial relationships that could be construed as a potential conflict of interest.

## Publisher’s note

All claims expressed in this article are solely those of the authors and do not necessarily represent those of their affiliated organizations, or those of the publisher, the editors and the reviewers. Any product that may be evaluated in this article, or claim that may be made by its manufacturer, is not guaranteed or endorsed by the publisher.
