# Supplementary material for: Identification of pyroptosis-related subtypes and establishment of prognostic model and immune characteristics in asthma
Source: Front Immunol. 2022 Jul 28;13:937832. doi: 10.3389/fimmu.2022.937832 (PMC9368761; doi:10.3389/fimmu.2022.937832)
Supplement: Supplementary file 1 [file DataSheet_1.pdf]

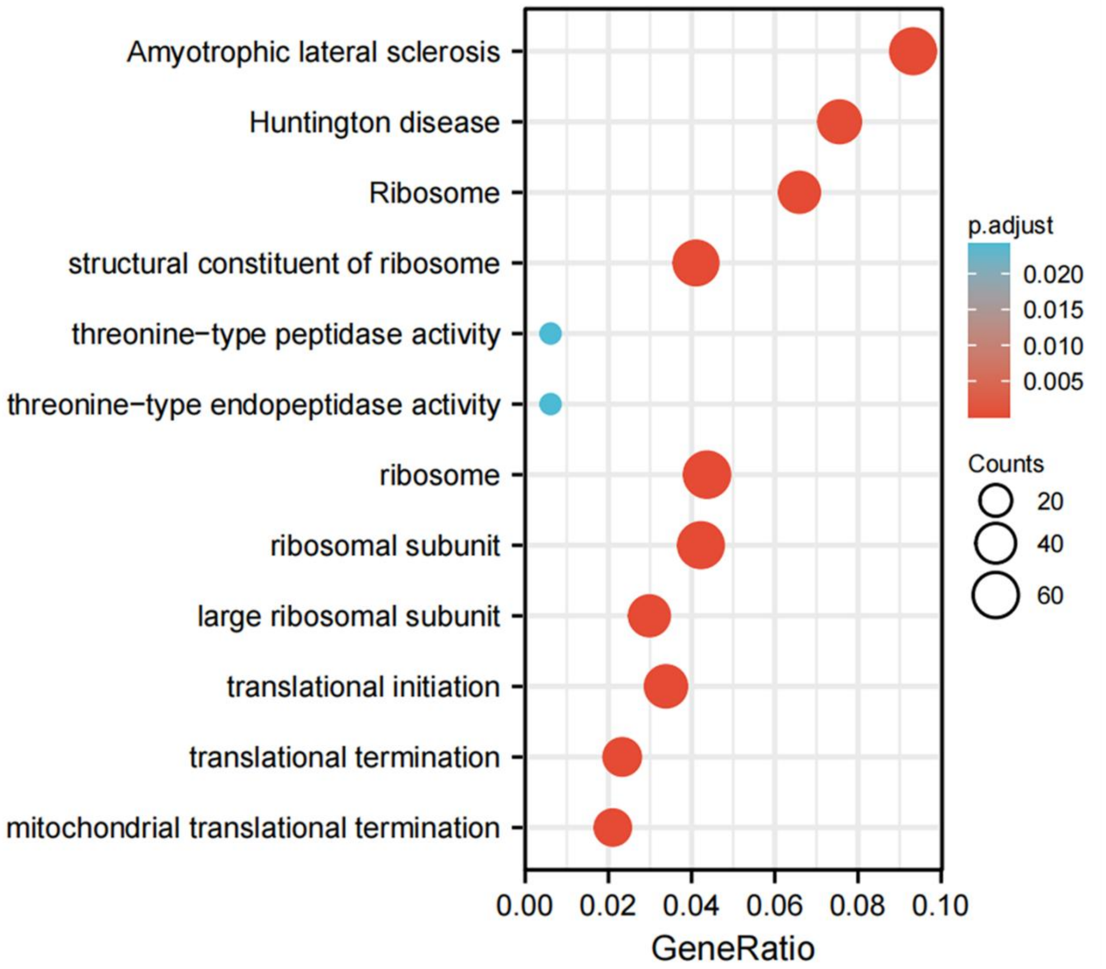

FIGURE S1 GO and KEGG enrichment analysis of DEGs between C1 and C2 subtypes.

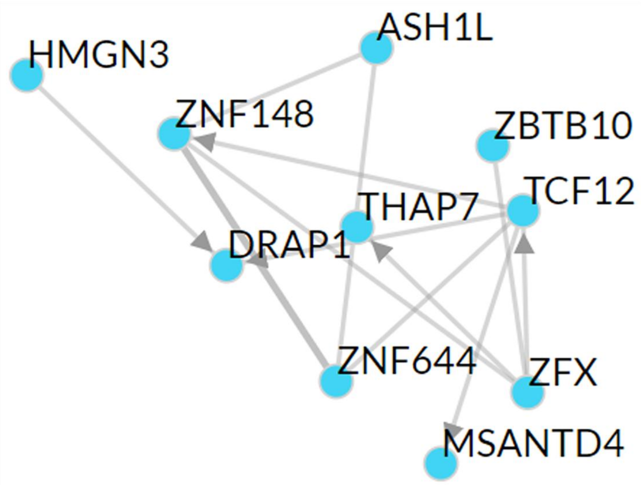

FIGURE S2 Transcription factors that regulate the expression of genes represented by the MEpink4 module and their interactions.

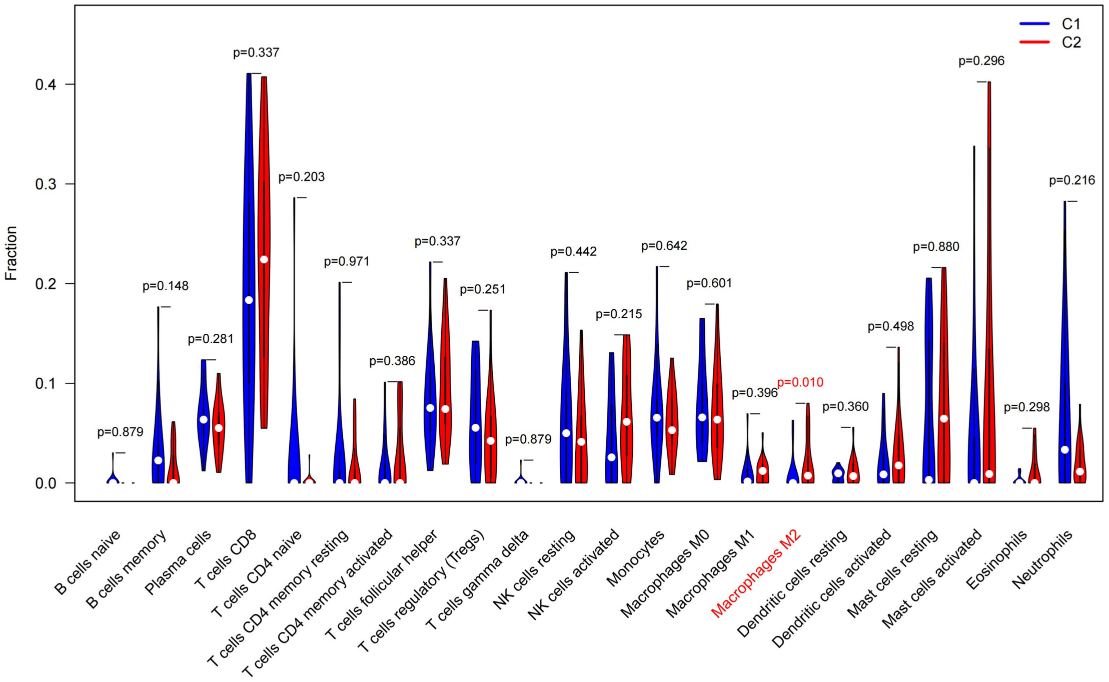

FIGURE S3 The relative contents of 22 kinds of immune cells in C1 and C2 subtypes.

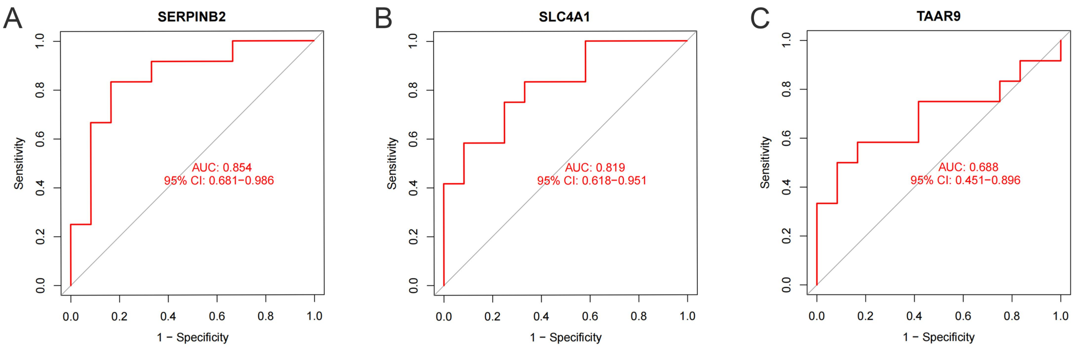

FIGURE S4 The ROC curve of the key genes in the model. (A) ROC curve for verifying the efficacy of SERPINB2 gene diagnosis. (B) ROC curve for verifying the efficacy of SLC4A1 gene diagnosis. (C) ROC curve for verifying the efficacy of TAAR9 gene diagnosis.

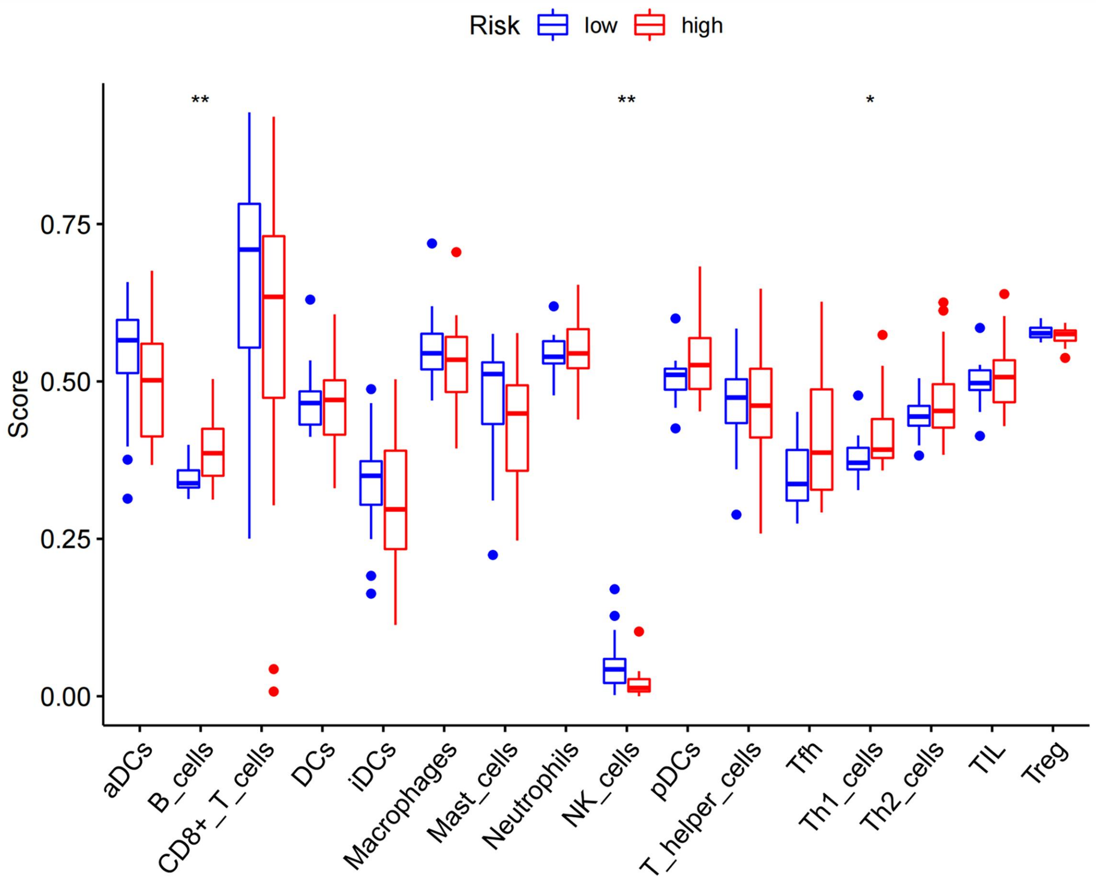

FIGURE S5 Differences in the abundance of infiltrating immune cells in the immune microenvironment between high- and low-risk groups.

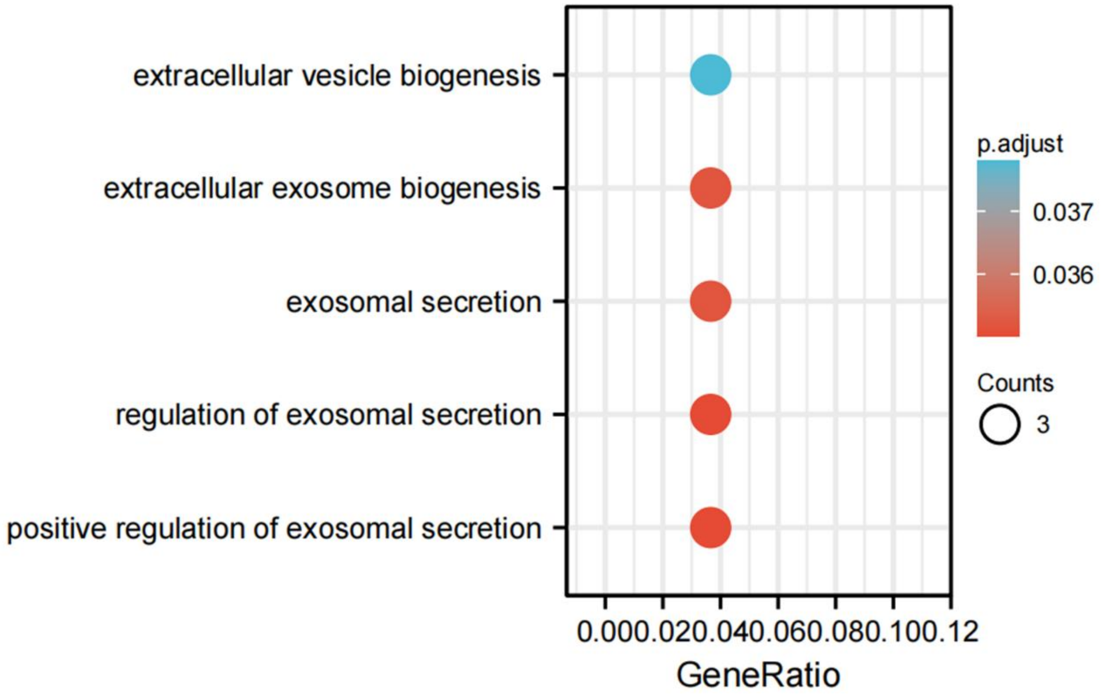

FIGURE S6 Functional enrichment analysis of DEGs between high risk group and low risk group.
